# Diet as prophylaxis and treatment for venous thromboembolism?

**DOI:** 10.1186/1742-4682-7-31

**Published:** 2010-08-11

**Authors:** David K Cundiff, Paul S Agutter, P Colm Malone, John C Pezzullo

**Affiliations:** 1 333 Orizaba Avenue, Long Beach, CA 90814, USA; 2 Theoretical Medicine and Biology Group, 26 Castle Hill, Glossop, Derbyshire, SK13 7RR, UK; 3 129 Viceroy Close, Birmingham, B5 7UY, UK; 4 Department of Medicine, Georgetown University, Washington, DC, USA

## Abstract

**Background:**

Both prophylaxis and treatment of venous thromboembolism (VTE: deep venous thrombosis (DVT) and pulmonary emboli (PE)) with anticoagulants are associated with significant risks of major and fatal hemorrhage. Anticoagulation treatment of VTE has been the standard of care in the USA since before 1962 when the U.S. Food and Drug Administration began requiring randomized controlled clinical trials (RCTs) showing efficacy, so efficacy trials were never required for FDA approval. In clinical trials of 'high VTE risk' surgical patients before the 1980s, anticoagulant prophylaxis was clearly beneficial (fatal pulmonary emboli (FPE) without anticoagulants = 0.99%, FPE with anticoagulants = 0.31%). However, observational studies and RCTs of 'high VTE risk' surgical patients from the 1980s until 2010 show that FPE deaths without anticoagulants are about one-fourth the rate that occurs during prophylaxis with anticoagulants (FPE without anticoagulants = 0.023%, FPE while receiving anticoagulant prophylaxis = 0.10%). Additionally, an FPE rate of about 0.012% (35/28,400) in patients receiving prophylactic anticoagulants can be attributed to 'rebound hypercoagulation' in the two months after stopping anticoagulants. Alternatives to anticoagulant prophylaxis should be explored.

**Methods and Findings:**

The literature concerning dietary influences on VTE incidence was reviewed. Hypotheses concerning the etiology of VTE were critiqued in relationship to the rationale for dietary versus anticoagulant approaches to prophylaxis and treatment.

Epidemiological evidence suggests that a diet with ample fruits and vegetables and little meat may substantially reduce the risk of VTE; vegetarian, vegan, or Mediterranean diets favorably affect serum markers of hemostasis and inflammation. The valve cusp hypoxia hypothesis of DVT/VTE etiology is consistent with the development of VTE being affected directly or indirectly by diet. However, it is less consistent with the rationale of using anticoagulants as VTE prophylaxis. For both prophylaxis and treatment of VTE, we propose RCTs comparing standard anticoagulation with low VTE risk diets, and we discuss the statistical considerations for an example of such a trial.

**Conclusions:**

Because of (a) the risks of biochemical anticoagulation as anti-VTE prophylaxis or treatment, (b) the lack of placebo-controlled efficacy data supporting anticoagulant treatment of VTE, (c) dramatically reduced hospital-acquired FPE incidence in surgical patients without anticoagulant prophylaxis from 1980 - 2010 relative to the 1960s and 1970s, and (d) evidence that VTE incidence and outcomes may be influenced by diet, randomized controlled non-inferiority clinical trials are proposed to compare standard anticoagulant treatment with potentially low VTE risk diets. We call upon the U. S. National Institutes of Health and the U.K. National Institute for Health and Clinical Excellence to design and fund those trials.

## Two accounts of the etiology of DVT and VTE

The consensus view that DVT and VTE are hematological disorders arose shortly after the Second World War and had become the new orthodoxy by the early 1960s. It still dominates research and practice in the field. Essentially, this consensus added 'hypercoagulability' to the 'stasis' and 'vessel wall injury' thesis of Hunterian pathophysiology, generating a set of loosely-defined terms that was misleadingly ascribed to Virchow [1,2]. In support of that consensus, at least some inherited and acquired thrombophilias ('hypercoagulability conditions') appear to increase the incidence of VTE, though this may indicate that thrombophilias aggravate rather than cause the disease. Moreover, there is an argument [3] that the so-called 'Virchow's triad' constitutes a useful rule of thumb for managing patients. Strikingly, however, the consensus view arose when anticoagulant therapy for thrombosis patients was becoming popular [4] and has developed along with such therapy and with the subsequent deployment of thrombolytic agents [1,2]. It seems integral with the pharmaceutical approach to DVT/VTE prophylaxis and treatment.

An alternative account of the etiology of DVT, the valve cusp hypoxia hypothesis (VCHH), is rooted in the tradition of thought and practice initiated by Hunter and traceable from Harvey through Virchow, Lister, Welch and a number of early 20^th ^century investigators [1,2]. According to the VCHH, DVT may occur wherever sustained non-pulsatile (streamline) venous blood flow leads to suffocating hypoxemia in the valve pockets, resulting in hypoxic injury to and hence death of the inner (parietalis) endothelium of the cusp leaflets. This injury activates the elk-1/egr-1 pathway, which initiates many responses of endothelial cells to hypoxia and activates chemoattractant and procoagulant factors [5]. (Briefly: elk-1 is a receptor tyrosine kinase stimulated by hypoxia; it phosphorylates the zinc-finger transcription factor early growth response-1 (egr-1), which then activates downstream genes encoding factors directly or indirectly involved in blood coagulation.) When normal pulsatile blood flow is restored, however transiently, leukocytes and platelets are attracted by these factors and inevitably re-enter the lately-affected valve pockets and marginate and sequestrate at the site of injury, the inner/parietal surfaces of the valve cusps, whereupon local blood coagulation (semi-solidification) is likely to be initiated.

Any subsequent period of non-pulsatile flow may kill the accumulated blood cells marginated on the dying or dead valve pocket. These dead cells may then form the core of a nascent thrombus. If periods of non-pulsatile and pulsatile flow continue to alternate, serial deposition of white cells and fibrin may ensue, resulting in the characteristic 'Lines of Zahn' morphology of a venous thrombus. Subsequently, only the blood cells on the outermost layer of a thrombus are living.

The VCHH explains many of the recognized risk factors for DVT and accounts for the morphology of thrombi. It also predicts that venous thrombi will readily embolize, because the area of endothelium to which they are anchored, the valve cusp parietalis, has become necrotic so it may be readily detached by the flow of blood past the obstruction.

Compared with the Virchow's triad hypothesis of DVT etiology, the VCHH better explains what appears to be a marked reduction in the incidence of hospital-acquired VTE (see below) following the introduction of early mobilization of post-operative patients and the widespread use of mechanical methods for maintaining pulsatile leg vein blood flow (e.g., flexion and extension of the ankles, support hoses, and intermittent pneumatic pressure leg devices). According to the VCHH, drugs that inhibit or 'kill' any part of the coagulation process might *slow the progression *of established DVTs but would be *ineffective in preventing the initiation *of thrombi.

## Problems with anticoagulant treatment for VTE

### Bleeding

Regarding patients treated for VTE with standard anticoagulants, a recent meta-analysis of published RCTs showed major and fatal bleeding rates of 1.8% and 0.2%, respectively [6]. Older cohort studies report up to triple these rates [7-9]. Applying the range of reported fatal bleeding rates for VTE treatment (0.2% - 0.6%) to an estimated 300,000-1.2 million people treated for VTE worldwide per year (about half in the USA [10]), 600-7,200 people per year suffer fatal bleeds from VTE anticoagulant treatment. There are many more non-fatal major bleeds, some of which are permanently debilitating.

Anticoagulant prophylaxis for surgical patients increases the risk of major bleeding [11]. VTE prevention trials report markedly different rates of major bleeding despite similar patient populations and doses and durations of anticoagulant prophylaxis. For instance, major bleeding with enoxaparin reportedly ranged from 0.1% to 3.1% in hip arthroplasty trials and from 0.2% to 1.4% in knee arthroplasty trials. If surgical-site bleeding is included in the definition of major bleeding, the reported rates have been about 10-fold higher [12]. Major bleeding adversely affects overall mortality. In a meta-analysis of trials comparing fondaparinux with LMWHs or placebos (major bleeding incidence overall = 2.4%), the risk of death by 30 days was 7-fold higher among patients with compared to those without a major bleeding event (8.6% versus 1.7%) [13]. If the major bleeding is considered the cause of the higher death rate, 6.9% of deaths in patients with major bleeds may be attributed to the bleeding (8.6% - 1.7% = 6.9%). Consequently, deaths of about 0.166% of anticoagulated patients are arguably attributable to bleeding (0.069 × 0.024 = 0.00166). Given that at least 12 million medical and surgical patients worldwide receive prophylactic anticoagulants per year [14,15], this means that approximately 20,000 people may die each year from complications of bleeding from prophylactic anticoagulants (0.00166 × 12 million = 19,872); many more may suffer the consequences of hypovolemia.

### Efficacy

Anticoagulant therapy for VTE became established as the standard of care in the 1940s and 1950s before randomized trials were considered necessary to prove efficacy and safety. A very small RCT comparing anticoagulants versus placebo for people with clinical diagnoses of PE published in 1960 [4] has been used to justify anticoagulant therapy. However, by current scientific standards, this study is highly flawed [10,16].

In 1962 when the U.S. Food and Drug Administration began requiring randomized controlled clinical trials (RCTs) showing efficacy before approving drugs, anticoagulation treatment of VTE was 'grandfathered in' with no rigorous efficacy trials ever required. Only three small methodologically rigorous RCTs of patients with DVTs [17-19] have compared standard anticoagulants with placebos or non-steroidal anti-inflammatory drugs. Combining the data from these trials, 6/66 patients receiving standard heparin and vitamin K inhibitors died and 1/60 unanticoagulated patients died [10]. Consequently, standard anticoagulant treatment for VTE cannot be considered evidence-based to be effective [10,20].

### Anticoagulant prophylaxis of 'high VTE risk' patients may increase fatal pulmonary emboli (FPE) due to 'rebound hypercoagulation'

Goldhaber and colleagues tracked the incidence of developing DVT or PE during or up to 30 days after hospital discharge in about 80,000 patients admitted over a two year period in Boston's Brigham and Women's Hospital. Out of 384 patients with hospital-acquired VTE, 318 (82.8%) were potential candidates for prophylaxis (i.e., they had ≥2 VTE risk factors). Of prophylaxis candidates, 170 (53%) of those with hospital-acquired VTE had received anticoagulants [21]. To estimate the influence of prophylactic anticoagulants in this study, we can use Goldhaber's USA-wide estimates of hospitalized patients that are at 'high VTE risk' -- 32% [14] or 25,600/80,000 in the Brigham and Women's Hospital study -- and the proportion of those at VTE risk who receive anticoagulant prophylaxis -- 50% [14] or 12,800/25,600 in this study. According to these estimates, 'high VTE risk' patients receiving anticoagulants in this population had a non-significant trend toward a higher incidence of VTE (OR = 1.15, 95% CI = 0.92 - 1.44) [22].

More importantly in this chart study, out of 13 deaths attributed to hospital-acquired FPE, 12 had received anticoagulant prophylaxis [21]. As above, assuming that 32% of the hospitalized patients were at risk for VTE and that 50% of all patients at risk for VTE received anticoagulants, anticoagulation prophylaxis was associated with a 12-fold *increase *in hospital-acquired FPE (OR: 12.0; 95% CI, 1.6-92) [22].

An autopsy study by Lindblad and colleagues [23] from Malmo, Sweden corroborated the Goldhaber study. From a population of 31,238 post-operative patients from the 1980s, it found that 27/30 patients with autopsy-proven FPE had received post-op prophylactic anticoagulants. The authors did not report the proportion of 'high VTE risk' surgical patients in their hospital receiving anticoagulant prophylaxis. To provide an approximation of the degree of increased risk of FPE related to anticoagulant prophylaxis in this autopsy study from a defined clinical population, we can conservatively assume that all Malmo surgical patients had 'high VTE risk' and again use Goldhaber's estimate that about 50% of those at risk received anticoagulant prophylaxis [14]. This translates to about 15,619 patients with anticoagulants and the same number without. Compared with patients not receiving anticoagulant prophylaxis, the Lindblad autopsy data show the estimated FPE rate in anticoagulated patients is nine-fold higher (OR: 9.0; 95% CI, 2.7-29.6).

Since many Malmo surgical patients would have been at 'low VTE risk' and fewer than 50% of those at 'high VTE risk' may have received anticoagulants in the 1980s, the FPE rate associated with anticoagulant prophylaxis could well have been considerably higher. Combining the FPE data from Goldhaber and Lindblad yields a very conservative estimated increased FPE risk associated with anticoagulant prophylaxis of 9.75 fold (OR, 9.75; 95% CI, 3.5 - 27.3). Combining these studies, 35/43 cases can be attributed to 'rebound hypercoagulation' (i.e., 39/43 FPE patients had received anticoagulation prophylaxis versus 4/43 with no anticoagulation: 39 - 4 = 35).

Surgery is associated with a substantial systemic and local activation of the coagulation and fibrinolytic systems. Post-operative prophylactic anticoagulants significantly mitigate the stimulation of these systems. However, following the discontinuation of prophylactic anticoagulants, a second wave of activation of markers of the coagulation and fibrinolytic systems continues for up to 35 days after surgery (e.g., plasma TAT and D-dimer [24]). Cundiff has suggested that 'rebound hypercoagulation' after stopping anticoagulants causing restimulation of coagulation and fibrinolysis may account for this marked increase in FPE risk associated with anticoagulant treatment [25] and prophylaxis [26]. Given that at least 12 million medical and surgical patients worldwide receive prophylactic anticoagulants per year [14,15], an estimated 5,000 to 40,000 people per year die of 'rebound hypercoagulation' (i.e., 12,000,000 (hospitalized people/year with anticoagulant prophylaxis) × 35/28,419 (excess risk for fatal PE per Goldhaber and Lindblad studies) = 14,779; 95% CI, 5,305 - 41,381).

While in the Goldhaber study 11/13 (85%) of FPE cases were in medical ward patients and only 2/13 were in surgical patients, the larger Lindblad study included anticoagulation prophylaxis data only on surgical patients. In the Lindblad study, 113 patients had PE as the principal cause of death, of which 83/113 (73%) were medical patients and 30/113 were post-operative. Lindblad did not report the anticoagulant prophylaxis status of the medical FPE patients. Since anticoagulated medical patients are about 50 times more likely than surgical patients to have FPE (Tables 1 and 2), the actual number of anticoagulated patients with FPE due to 'rebound hypercoagulation' is likely much higher than derived from combining these two autopsy studies because of the disproportionately high number of surgical patients.

**Table 1:** FPE incidence VTE observational studies and RCTs in medical patients from the 1980s to 2000s

Author	FPE incidence no anti-coagulation	FPE incidence with anti-coagulation
Mahé [89]	17/1,244	10/1,230

Alikhan [87]	467/9,491	431/9,349

Cohen [90]	5/414	0/425

Testroote [91]	0/454	0/442

Bergmann [92]	17/1,244	10/1,230

Bergmann [93]	NA	2/439

Fraisse [94]	0/114	1/109

Turpie [95]	0/650	0/635

	506/13,611 (3.7%)	453/13,859 (3.3%)

**Table 2:** FPE incidence in surgical patients: VTE observational studies and RCTs in the 1980s to 2000s

Population of surgical patients)	Author	FPE incidence no anti-coagulation	FPE incidence with anti-coagulation
general surgical	Kosir [96]	0/70	0/38

general surgical	Kosir [97]	1/68	0/68

general surgical	Rasmussen [98]	1/405	0/388

total general surgical		2/543 (0.37%)	0/494 (0%)

			

orthopedic surgical	Sasaki [99]	0/38	0/38

orthopedic surgical	Bi [100]	0/35	0/35

orthopedic surgical	Goel [101]	0/111	0/127

orthopedic surgical	Agarwal [102]	0/131	0/166

orthopedic surgical	Eriksson [103]	NA	0/1,587

orthopedic surgical	Eriksson [104]	NA	0/1,464

orthopedic surgical	Heit [105]	NA	1/594

orthopedic surgical	Eriksson [106]	NA	0/133

orthopedic surgical	Francis [107]	NA	0/2,285

orthopedic surgical	Eriksson [108]	NA	1/2,056

orthopedic surgical	Turpie [109]	NA	5/7,211

orthopedic surgical	Ramos [110]	0/262	0/267

orthopedic surgical	Ginsberg [111]	NA	1/1,896

orthopedic surgical	Agnelli [112]	NA	0/507

orthopedic surgical	Turpie [113]	NA	0/613

orthopedic surgical	Colwell [114]	NA	0/1,838

orthopedic surgical	Eriksson [115]	NA	1/1,872

orthopedic surgical	Eriksson [116]	NA	2/2,835

orthopedic surgical	Eriksson [117]	NA	1/2,788

orthopedic surgical	Colwell [118]	NA	3/2,299

total orthopedic surgical		0/577 (0%)	15/29,291 (0.051%)

			

unspecified surgical	Rosenzweig [119]	0/4,705	NA

unspecified surgical	Nurmohamed [120]	NA	11/8,172

total unspecified surgical		0/4,705 (0%)	11/8,172 (0.135%)

			

surgical totals		2/5,825 (0.034%)	26/37,957 (0.068%)

### Marked reduction in FPE risk over time unrelated to anticoagulants

In the 1960s and 1970s, FPE in trials of post-op surgical patients without anticoagulant prophylaxis averaged 0.99% while FPE rates in anticoagulated patients averaged 0.31% (Table 3). Since about 1980, prompt ambulation of post-op patients and other non-drug VTE prophylaxis measures (e.g., mechanical prophylaxis of lower limbs) have been widely implemented. Recent observational studies and RCTs of surgical patients at VTE risk both not receiving and receiving prophylactic anticoagulants show a somewhat reduced VTE incidence and a markedly lower FPE frequency than seen in studies from the 1960s and 1970s (see Tables 2 and 4).

**Table 3:** FPE incidence in surgical patients in the 1960s and 1970s

Population (medical, surgical, etc.)	Author	FPE incidence no anti-coagulation	FPE incidence with anti-coagulation
general surgical	Clagett [27]	48/5,547 (0.87%)	19/6,845 (0.28%)

orthopedic surgical	Collins [29]	15/801 (1.87%)	5/826 (0.61%)

total surgical		63/6,348 (0.99%)	24/7,671 (0.31%)

**Table 4:** FPE incidence in autopsy studies from the 1980s to 1990s

Population (medical, surgical, etc.)	Author	FPE incidence no anti-coagulation	FPE incidence with anti-coagulation
surgical	Lindblad [23]	3/15,619	27/15,619

medical and surgical	Goldhaber* [21]	1/12,800	12/12,800

		4/28,419 (0.014%)	39/28,419 (0.13%)

* 8/13 patients had autopsy confirmation

The data from the 1960s and 1970s, on which the evidence basis for anticoagulation prophylaxis of patients at high risk for VTE relies, do not pertain to 'high VTE risk' hospitalized patients in the 21^st ^century for eight reasons:

1. Very few of the subjects in the earlier studies received mechanical prophylaxis such as graded compression stockings, which are now the standard of care and have been shown in a meta-analysis of trials from the 1960s and 1970s to reduce VTE significantly more than low-dose heparin (VTE with low dose heparin: 23/173 (13.3%) versus VTE with compression stockings: 14/190 (6.8%), *P *= 0.04) [27]. In an RCT published in 1996 of VTE prophylaxis for neurosurgical patients comparing graded compression stockings alone with graded compression stockings plus LMWH, the LMWH plus stockings group had a significantly higher overall mortality (22/241 versus 10/244: p = 0.026) [28].

2. Post-operative and medical patients today become ambulatory much earlier than in the 1960s and 1970s, reducing FPE risk.

3. Probably because of #1 and #2 above, rates of FPE in 'high VTE risk' surgical patients without anticoagulant prophylaxis from the 1960s and 1970s are over 40 times the rates reported from more recent studies (63/6,348 (0.99%)) [27,29] (Table 3) versus 5/21,444 (0.023%) from Lindblad's post-op autopsy study (Table 4[23]) combined with a representative sampling of surgical anticoagulation prophylaxis RCTs (Table 2).

4. In studies from 1980 to 2010, the rate of FPE in surgical patients receiving anticoagulant prophylaxis (53/53,576 (0.10%), combining Table 4 Lindblad with Table 2 totals) is over four times higher than the FPE rate of recent unanticoagulated surgical patients (5/21,444 (0.023%), Table 4 Lindblad and Table 2 totals). This suggests that anticoagulant prophylaxis may now *increase *FPE.

5. Very few of the recent or old VTE prophylaxis RCTs (anticoagulant versus none) included FPE cases occurring after discontinuation of the anticoagulant and discharge from hospital, thereby missing those dying of 'rebound hypercoagulation'. In the Goldhaber chart study above, 45% of hospital-acquired VTE cases occurred in the 30 days after hospital discharge. On the basis of the Goldhaber and Lindblad studies [21,23] that included FPE occurring at least one month after stopping prophylactic anticoagulation, about 80% of FPE cases documented at autopsy in recent years appear to be due to 'rebound hypercoagulability' (i.e., 35/43, see above).

6. Owing to the high rate of FPE in unanticoagulated 'high VTE risk' patients in the 1960s and 1970s (0.99%) and even in those then receiving anticoagulant prophylaxis (0.31%), 'rebound hypercoagulability' related FPE in that previous era would have been missed. Relative to the FPE rates in the 1960s and 1970s, it occurred infrequently in the post-1980 Goldhaber and Lindblad studies (i.e., 0.12% (35/28,400), Table 4, or about 1/800 patients). However, since 1980 with markedly lower FPE rates in post-op patients generally (i.e., 0.034% without anticoagulation and 0.068% with anticoagulant prophylaxis, Table 2), we should be very concerned about missing a 0.12% estimated incidence of 'rebound hypercoagulation'-related FPE.

7. The FPE rates in medical patients in the 1960s and 1970s are not documented in anticoagulation versus no anticoagulation RCTs. From 1980 to 2010, medical patients have had up to 100 times the FPE rate of surgical patients and that rate is not reduced significantly by anticoagulant prophylaxis (i.e., no anticoagulation: 3.7% versus anticoagulated: 3.3%, Table 1). However, these medical patient trials record FPE only while patients are on anticoagulants and also potentially miss cases of FPE due to 'rebound hypercoagulation'.

8. A high proportion of patients with autopsy-verified FPE had underlying terminal illnesses (e.g., FPE rates in two large autopsy series: 95% (169/178 [30]) and 96.5% (1,867/1,934 [31])). Since surgeons try to avoid performing elective operations on terminally ill people and medical services frequently care for terminally ill patients, the low FPE rate in surgical RCTs and high rate in acute medical patients makes sense. Out of the total group of 'high VTE risk' patients, those undergoing prolonged bed rests due to cancer, heart failure, or other organ failure may be particularly prone to FPE despite being on prophylactic anticoagulants and, additionally, due to 'rebound hypercoagulation'.

Given (1) the incidence of major and fatal bleeding from anticoagulants for prophylaxis and treatment of VTE, (2) the efficacy data for both that have been called into question, and (3) the evidence for previously unrecognized and largely uncounted deaths from 'rebound hypercoagulability'; reconsideration of the evidence-basis of anticoagulants for treatment and prophylaxis of VTE is in order.

## Diet and VTE

Although therapeutic diets are widely suggested for prophylaxis and treatment of arterial cardiovascular disease, healthy nutrition as an approach to prophylaxis and treatment of VTE has never been officially recommended. Acting U.S. Surgeon General Dr. Steven Gaston noted in his call to action to prevent VTE that the "Longitudinal Investigation of Thromboembolism Etiology (LITE) " study [32] found a diet with more fruits, vegetables, and fish, and less red and processed meat to be associated with a lower VTE incidence. He suggested further studies on the impact of diet and other lifestyle changes regarding VTE [33].

Data about the relationship of diet to VTE risk come from:-

• historical observations about the incidence of FPE under wartime conditions, including food rationing, in early 20^th ^century European cities;

• prospective observational studies of diet and lifestyle factors associated with VTE;

• case-control studies of VTE patients looking at lipid profiles, inflammation markers, and coagulation variables;

• comparisons among people on various diets regarding lipid profiles, inflammation markers, and coagulation variables.

### Historical data

In Norway from 1940 to 1944, intake of meat, whole milk, cream, margarine, cheese, eggs, and fruit decreased while people increased their intake of fish, cod liver oil, skimmed milk, whole grain bread, potatoes, and fresh vegetables. The rate of post-operative VTE decreased markedly during the Second World War in Norway followed by a marked increase after the war [34].

During the Second World War, people in Norway, Sweden, Switzerland, Germany, Finland, and Denmark had significantly reduced intake of food from animal sources. However, only Denmark showed no decrease in vascular disease mortality. In Denmark alone, there was no significant reduction in consumption of dairy fats and eggs [35].

**Figure 1:**
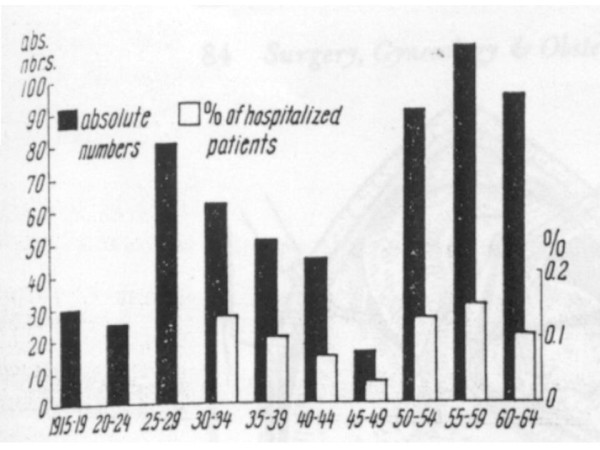
**Fatal PE from 1915 to 1964 in Heidelberg, Germany **[36]. Absolute numbers of patients with autopsy-proven FPE in black, and percentage of in hospital patient deaths related on autopsy to PE in white. Reproduced from Linder et al. [88].

The autopsy incidence of FPE over time in Heidelberg, Germany showed a clear relationship between pulmonary embolism and wartime conditions. The lowest incidence of FPE, expressed as a percentage of all hospitalized patients, was registered during the post-Second World War years with a relative and absolute minimum between 1945 and 1949. The 1947 value (0.04%) was lower than 1932 (0.45%) or 1955 (0.38%) [36] (Fig. 1).

**Figure 2:**
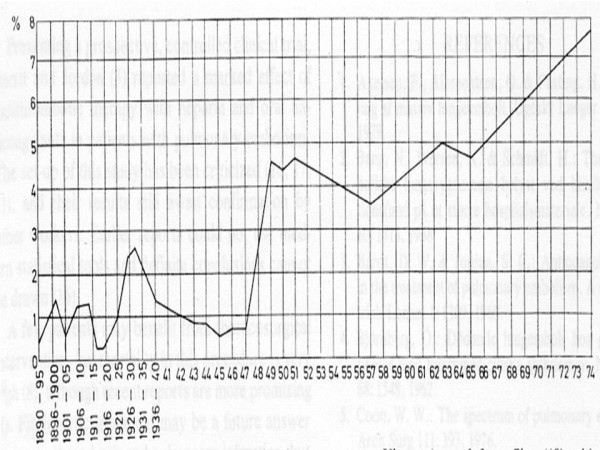
**Vienna, Austria percentages of autopsies with fatal PE (Quoted by Nielsen from Sigg **[30,36]).

In Vienna after the First World War, FPE accounted for less than 0.5% of deaths versus 2.5% in the early 1930s. Again, in the late 1940s, incidence of FPE at autopsy was <1% versus almost 8% by the early 1970s [30] (Fig. 2).

These historical studies have limitations but suggest that the high-complex-carbohydrate, low-fat diet associated with war-time food rationing and perhaps increased exercise may have markedly reduced the tendency to form thrombi and/or lessened the consequences of those that do form. Judging from the autopsy data, the effects of these lifestyle influences on VTE risk had a rapid onset and offset, and wartime conditions afforded substantial protection against VTE, especially FPE.

### Prospective observational studies of diet and lifestyle factors associated with VTE

In the "Longitudinal Investigation of Thromboembolism Etiology (LITE) " prospective study, hazard ratios (95% CIs) of VTE incidence across quintiles of fruit and vegetable intake were: 1.0 (reference: lowest quintile), 0.73 (0.48 to 1.11), 0.57 (0.37 to 0.90), 0.47 (0.29 to 0.77), and 0.59 (0.36 to 0.99) with *P*trend = 0.03 [32]. The fruit and vegetable intake in the lowest quintile, 2.0 servings per day, was far less than recommended by the United States Center for Disease Control (i.e. >5 servings per day for most people) [37]. The highest quintile averaged 6.7 servings per day. Meat intake was a predictor of VTE risk in LITE (HRs of VTE across quintiles of red and processed meat intake--1.0 (lowest quintile), 1.24 (0.78 to 1.98), 1.21 (0.74 to 1.98), 1.09 (0.64 to 1.87), and 2.01 (1.15 to 3.53) with the *P*trend = 0.02 [32]). Since fruit/vegetable intake in LITE correlated negatively with meat intake (r = - 0.28), the two most influential dietary variables may have acted synergistically on VTE risk.

In contrast, the Iowa Women's Health Study (IWHS) [38], to date the only other large prospective study of diet related to VTE risk, found no associations of VTE risk with intake of fruits/vegetables, meat, fish, or other foods. It also found no significant associations with dietary patterns or individual nutrients. The IWHS found an association of daily alcohol consumption with lowered VTE risk, whereas LITE only affirmed that adjusting for alcohol consumption did not diminish the strength of the correlations between diet and VTE [32]. The following differences between the LITE and IWHS studies may account for the discrepancies:

• Only women were surveyed in the IWHS (99% white women); the LITE study included relatively fewer women (55%) and more non-whites (27%).

• LITE's dietary assessment had an interviewer-administered food frequency questionnaire (FFQ), considered more precise than the self-administered IWHS questionnaire [32].

• While the IWHS FFQ assessment was done only once and related to the subsequent 19 year (mean 13 year) follow-up for VTE incidence, the LITE FFQ assessment was done twice, six years apart (r ranged from 0.49 to 0.56 between the two assessments). This allowed the LITE diet assessment to be based on a cumulative average dietary intake. The designers of the FFQs used in both studies found that using the cumulative averages, in general, yielded stronger associations than utilizing either baseline diet or the most recent diet alone [39].

Therefore, the LITE study findings relating fruit and vegetable intake to reduced VTE risk and meat intake to increased VTE risk have more scientific validity than the IWHS finding of no dietary factor influences on VTE. The LITE findings are also consistent with the historical observations (above).

### VTE risk related to lipids, inflammation, and hemostatic parameters

A meta-analysis of VTE case-control studies found that VTE patients had significantly lower HDL cholesterol levels and higher triglycerides but no differences in total cholesterol or LDL-cholesterol [40]. Prospective studies of associations between lipid levels and VTE risk are conflicting but possibly suggest that VTE risk is reduced when serum levels of HDL cholesterol are higher and triglyceride lower [41].

In LITE, VTE was not correlated with C-reactive protein or white cell count [42]. However, LITE did not differentiate between idiopathic versus secondary VTE cases. Luxembourg and colleagues reported a case-control study comparing idiopathic VTE with risk-associated VTE patients (post-op, etc.) and controls. Idiopathic VTE patients had significantly higher levels of high-sensitivity C-reactive protein (hs-CRP) than secondary VTE patients (mean 1.8 versus 1.5, P = 0.05), who had significantly higher levels than controls (mean 1.5 versus 1.2, P = 0.02) [43].

Case-control and prospective studies show correlations of VTE with serum levels of factor VIII [43-47]. The VTE case control study by Luxembourg and colleagues found significantly higher fibrinogen levels in patients with idiopathic VTE than those with risk-associated VTE (median: 331 versus 299 mg/dl, p = 0.004) [43]. Controls had levels similar to risk-associated VTE patients (median: 302 mg/dl). In LITE, factor VIII levels and von Willebrand factor levels correlated significantly with VTE (P for trend in quartiles <0.0001 for both) but fibrinogen levels did not. LITE did not assess platelet aggregation or indices of fibrinolysis.

## Alcohol and VTE

The Iowa Women's Health Study reported that daily alcohol imbibers suffered fewer episodes of VTE. This finding is questionable, since a single assessment of alcohol intake was related to VTE incidence over the ensuing 19 years [38]. The LITE study found that alcohol consumption neither increased nor decreased VTE risk [32].

There is no other prospective epidemiological evidence linking alcohol use with either increased or decreased risk of VTE.

## Fish intake and VTE

In LITE, fish intake was deemed protective against VTE on the basis of a comparison of VTE incidence between the first quintile of fish consumption and the sum of the four subsequent quintiles: 1.0 (first quintile) 0.58 (0.37-0.90) 0.60 (0.39-0.92) 0.55 (0.35-0.88) 0.70 (0.44-1.10), Ptrend= 0.30). This post hoc comparison is dubious because fish consumption correlated positively with intake of fruits/vegetables (r = 0.27, P < 0.001) [32]. The correlation between fish and meat consumption was not reported. In an analysis of the Diabetes Control and Complications Trial database by Cundiff and colleagues [48], long chain omega-3 fatty acid intake (a marker for fish consumption) correlated inversely with percentage of calories from saturated fatty acids (r = -0.21, *P *< 0.0001), and directly with dietary fiber intake (g/1,000 kcal) (r = 0.20, *P *< 0.0001), suggesting that fish eating is correlated with a more plant-based than animal-based diet. In a Greek study of diet in people with and without acute coronary syndromes [49], fish intake was associated with consumption of:

• red meat - inversely related in patient and control groups (*P <*0.001 for both);

• vegetables - directly correlated (*P <*0.001 for both);

• fruit - directly correlated (*P <*0.001 for both); and

• legumes - directly correlated (*P <*0.001 for both).

Because fish intake is confounded with other healthy dietary choices in the LITE database and other studies, fish consumption does not appear to be an independent protective factor for VTE. The possibility may nevertheless merit further study taking account of the confounding variables.

## Diets to consider for lowering VTE risk

Low VTE risk diets (i.e., high in fruits and vegetables and low in red and processed meats) to consider as the experimental arm of non-inferiority randomized trials evaluating standard anticoagulants for prophylaxis and treatment of VTE are as follows:

### American Heart Association (AHA) step 1 and step 2 diets

AHA step 1 and step 2 diets recommend plenty of fruits and vegetables, lean meat and two servings of fish per week [50]. A meta-analysis of randomized trials of these diets versus regular diets (27 trials with more than 30,000 patient years of follow-up) shows no significant reduction of overall mortality (RR: 0.98, 95% CI 0.86 to 1.12), or cardiovascular disease mortality (RR: 0.91, 95% CI 0.77 to 1.07) [51,52].

There is no evidence that the AHA step 1 or step 2 diets would reduce VTE risk any more than overall cardiovascular risk, so they would not be good low VTE risk diet candidates.

### Mediterranean diet (MD)

While the MD does not significantly benefit serum lipids, blood pressure, or body mass index, it reduced overall cardiovascular disease risk by 70% in the "Lyon Diet Heart Study " [53].

Studies in Table 5 suggest that the MD benefits markers of coagulation, inflammation, and cardiovascular disease risk.

**Table 5:** MD studies of serum markers of inflammation and coagulation

Author	Study Design	Population	Exposure variable	Outcome variable	Results
Esposito [76]	RCT	Metabolic syndrome patients	MD	1. Nutrient intake 2. endothelial function 3. lipid and glucose parameters 4. insulin sensitivity 5. hs-CRP 6. IL-6 7. IL-7 8. IL-18	With MD 1. hs-CRP decreased (P = 0.01) 2. IL6 decreased 3. (P = 0.01) 4. Endothelial function improved (P < 0.001) 5. lipid and glucose parameters improved (P < 0.001) 6. decreased insulin resistance (P < 0.001)

Mezzano [77]	RCT	Healthy volunteers	MD versus high fat diet	Fat content Fibrinogen factor VIIc factor VIIIc protein S	Fat content - MD: 27.3% - HFD: 39.9% With MD Fibrinogen reduced (P = 0.03) factor VIIc reduced (P = 0.034) factor VIIIc reduced (P = 0.0057) protein S increased (P = 0.013)

Antonopoulou [75]	Observa-tional	Healthy volunteers and type 2 DM patients	MD	platelet aggregation in response to platelet aggregating factor or thrombin	Platelet activity reduced in both groups

Chrysohoou [74]	Observa-tional	People in Greece	Adherence to MD comparing the highest and lowest tertile	CRP IL-6 Homocysteine WBCs	highest tertile participants averaged - 20% lower CRP levels (P = 0.015) - 17% lower interleukin-6 levels (P = 0.025) - 15% lower homocysteine levels (P = 0.031) - 14% lower white blood cell counts (P = 0.001) - 6% lower fibrinogen levels (P = 0.025)

### Vegetarian diets

Epidemiological studies show that people consuming vegetarian diets (lacto-ovo, lacto, ovo, or not otherwise specified (NOS)) and vegan diets (no meat, dairy, or eggs) have lower overall vascular disease incidence than omnivores [54-58].

A lacto-ovo vegetarian diet contains plenty of fruits and vegetables and no meat, largely conforming to the LITE prospective data regarding a low VTE risk diet [32]. However, the lack of decreased vascular disease risk in Denmark during and after the Second World War (see above) [35] might be considered a point in favor of a vegan diet.

Tables 6, 7, and 8 show representative studies of vegetarian and vegan diets related to VTE risk and inflammatory, lipid, and hematological markers, respectively, demonstrating trends favorable to these diets.

**Table 6:** Studies of vegetarian diets and serum markers of inflammation

Author	Study Design	Population	Exposure variable	Outcome variable	Results
Mezzano [70]	Case-control	52 Chilean subjects	Lacto or lacto-ovo Vegetarians v. Omnivores	CRP	NS

Chen [65]	Case-control	198 healthy Taiwanese subjects	Vegetarians (NOS) v. Omnivores	CRP	NS

Harvinder [121]	Case-control	47 USA subjects with CAD or CAD risk factors	Vegans v. Omnivores	CRP	Vegans had significantly lower levels of CRP

Kjeldsen-Kragh [64]	Case-control	53 Rheumatoid arthritis patients	Lacto vegetarians v. Omnivores	WBC count	Lacto vegetarians had significantly lower WBCs

Kjeldsen-Kragh [72]	Case-control	Rheumatoid arthritis patients	Vegetarians (NOS) v. Omnivores	WBC count	Vegetarians had significantly lower WBCs

Nazarewicz [62]	Case-control	22 vegetarian and 19 omnivore Pols	Vegetarians (NOS) v. Omnivores	WBC count	Vegetarians had significantly lower WBCs

Pongstaporn [122]	Case-control	178 vegetarian and 58 omnivore Thais	Vegetarians (NOS) v. Omnivores	WBC count	Vegetarians had significantly lower WBCs

Arm-strong [63]	Case-control	431 vegetarian and 131 omnivore Seventh-day Adventists	Vegetarians (NOS) v. Omnivores	WBC count	Vegetarian men but not women had significantly lower WBCs

Haddad [68]	Case-control	25 vegan and 20 omnivore Californians	Vegans v. Omnivores	WBC count	Vegans had significantly lower WBCs

Tungtrong-chitr [67]	Case-control	132 vegetarians and 47 omnivores from Thailand	Vegetarians (NOS) v. Omnivores	WBC count	NS

Malter [66]	Case-control	German male vegetarians and omnivores	Vegetarians (NOS) v. Omnivores	WBC count	NS

**Table 7:** Studies of vegetarian diets and serum lipid markers

Author	Study Design	Population	Exposure variable	Outcome variable	Results
Li [73]	Case-control	139 healthy male subjects aged 20-55 Melbourne	Vegetarians (NOS) v. Omnivores	ratios of triglycerides/HDL-cholesterol	Vegetarians had lower ratios of triglycerides/HDL-cholesterol

Chen [65]	Case-control	198 healthy Taiwanese subjects	Vegetarians (NOS) v. Omnivores	levels of total cholesterol and LDL-C	Vegetarians had lower levels of total cholesterol and LDL-C

**Table 8:** Studies of vegetarian diets and serum markers of coagulation

Author	Study Design	Population	Exposure variable	Outcome variable	Results
Li [73]	Case-control	139 healthy male subjects aged 20-55 in Melbourne	Vegetarians (NOS) v. Omnivores	factor VII activity	Lacto-ovo vegetarians had significantly lower plasma factor VII activity

Mezzano [70]	Case-control	52 Chilean subjects	Lacto or lacto-ovo Vegetarians v. Omnivores	PT, fibrinogen, factor Vc, factor VIIc, factor VIIIc, antithrombin III, protein S, plasminogen, protein C	Lacto-ovo vegetarians had significantly lower levels of fibrinogen, factor Vc, factor VIIc, factor VIIIc, antithrombin III, protein S, plasminogen, prothrombin, protein C

Pan [123]	Case-control	203 healthy Taiwanese age <30	60 vegetarians and 143 omnivores	PT, APTT, fibrinogen, factor VIIc, factor VIIIc, antithrombin III, plasminogen,	Vegetarian men did not differ from omnivore men. Women: factor VIIIc higher and APTT shorter in vegetarian women versus omnivore women

A study from Rotterdam assessed the relationship of dietary fat and fiber with coagulation factor VII in 3,007 elderly men and women subjects. Total fat and saturated fat intake were significantly associated with factor VIIc only in women. Fiber intake was inversely associated with factor VIIc in both men and women [59].

Mediterranean, vegetarian or vegan diets could all be reasonable choices for the experimental arm of non-inferiority trials with anticoagulants for prophylaxis and treatment of VTE. Given the challenges to patients in changing diets and the likelihood that RCTs would show that anticoagulation prophylaxis and treatment for VTE are themselves ineffective as suggested in the background section above, RCTs of VTE patients should allow considerable flexibility in the diet interventions so that they are not burdensome.

## Vitamin K intake and VTE risk

Studies on vitamin K and VTE risk or coagulation profiles are very limited, because there is no established role of vitamin K supplementation except in people who are deficient in vitamin K. Based on the hypothesis that vitamin K supplementation may protect against atherosclerosis, a placebo-controlled randomized trial evaluated the effect of phylloquinone supplementation on blood lipids, inflammatory markers, and fibrinolytic activity in postmenopausal women. No effect was seen on inflammatory or fibrinolytic markers and lipid markers worsened (i.e., increased triacylglycerols and decreased HDL-C) [60]. A systematic review of RCTs of vitamin K supplementation for preventing bone loss and fractures yielded 13 trials. None of the trials reported an increase in VTE or other adverse reactions [61]. Vitamin K status does not seem to be a significant factor in VTE risk.

## Effects of diet in relation to the VCHH

The simplest and most plausible mechanistic link between diet and DVT/VTE is through other aspects of lifestyle: people who eat unhealthy diets are likely to exercise less and remain sedentary for longer than those who eat healthy diets, exposing them to a greater risk of prolonged non-pulsatile venous blood flow and consequent valve pocket hypoxemia. However, subtler links between diet and venous thrombogenesis can be inferred from the VCHH.

Compared with subjects taking omnivorous diets, a non-vegan vegetarian diet is associated with a lower neutrophil count in some studies [62-64] though not all [65-67]. Subjects on vegan diets consistently show lower neutrophil counts [68,69]. Studies have provided mixed findings regarding platelet counts and function in vegetarians and vegans [62,68,70-73].

A reduced number of neutrophils would tend to attenuate the invasion of a hypoxically injured (necrotic) valve cusp endothelium by leukocytes, which according to the VCHH would militate against the events initiating thrombogenesis. This suggests a mechanism by which vegan diets could reduce the risk of VTE.

In that the MD has been found to decrease the white blood cell count [74], reduce platelet activity [75], decrease markers of inflammation [76], improve endothelial function [76], and lower factor VII and VIII levels [77]; it may be consistent with reduced neutrophil margination and sequestration on a hypoxically damaged valve cusp, associated with elevation of EDRF and non-elevation of p38 MAPK and thus with a decreased risk for venous thrombogenesis (see chapter 12 in [2]) [78].

## Randomized controlled non-inferiority trials of anticoagulants for VTE proposed

Given the risks of anticoagulation for VTE and new data concerning the current reduced risk of hospital-acquired VTE, researchers should consider non-inferiority randomized trials to test the efficacy and safety of anticoagulants for prophylaxis of VTE for medical and surgical patients. Likewise, given the lack of RCT evidence of efficacy of anticoagulant treatment of VTE, a non-inferiority RCT would be in order to test the efficacy and safety of anticoagulants for treatment of VTE.

To enlist researchers and patients in these proposed trials, the rationale must be clearly explained and appropriate experimental arms determined. Experimental arm options include using placebos or non-steroidal anti-inflammatory drugs (NSAIDs). However, a Cochrane review of anticoagulation treatment for VTE concluded that: "The use of anticoagulants is widely accepted in clinical practice, so a further RCT comparing anticoagulants to placebo could not ethically be carried out " [20]. An NSAID would have the advantage of being a weak antithrombotic with less bleeding risk than anticoagulants; however, epidemiological evidence suggests that NSAIDs may increase the risk of VTE [79].

Given the relationship between diet and VTE noted above, low VTE risk diet interventions should be explored as possible experimental arms in randomized non-inferiority trials testing the efficacy and safety of anticoagulants for prophylaxis and treatment of VTE.

If such trials yield positive results, they may be especially useful in developing countries, where there is a significant and increasing risk of DVT and VTE in medically ill patients but only a minority receives anticoagulant prophylaxis [80,81]. Practitioners in these countries recognize the economic and other difficulties of providing anticoagulants for all patients for whom they are not contraindicated and recommend careful risk stratification and education [80,82]. Education could include dietary advice. Interestingly, there is strong evidence that oral contraceptives currently used in developing countries entail a statistically significant risk of VTE, but only among women with high body mass indices [83,84]; again, this could indicate that dietary prophylaxis would be beneficial.

## Statistical considerations in designing non-inferiority trials

To provide convincing evidence for a change in standard-of-care treatment/prophylaxis for VTE, from anticoagulants to a lower-risk alternative like low-VTE-risk diet, a definitive clinical trial would have to accomplish two goals simultaneously:

1. demonstrate that the diet is significantly **safer **than anticoagulants with respect to hemorrhage, **and**

2. demonstrate that the diet is **non-inferior **to anticoagulants with respect to the prevention or amelioration of major complications of VTE.

This requires a safety/efficacy trial with **two co-primary endpoints**...

1. a primary **safety **endpoint -- an undesired hemorrhagic event, and

2. a primary **efficacy **endpoint --an undesired thromboembolic event, such as fatal or non-fatal PE, recurrent symptomatic PE, or symptomatic DVT.

The choice of the two co-primary endpoints is of utmost importance because these endpoints determine the sample size needed for the study -- there must be a sufficient number of each endpoint observed, so that the study will have sufficient statistical power to yield simultaneous significant outcomes in testing each of the two co-primary study hypotheses -- better safety *and *non-inferior efficacy for the low VTE risk diet, relative to the anticoagulants.

So the co-primary safety and efficacy endpoints must be **consequential **enough to provide convincing motivation to change medical practice, and **frequent **enough to yield a sufficiently powered study with a reasonable sample size. Of course, a set of secondary safety and efficacy endpoints would also be monitored and analyzed. For any given choice of endpoints, the study design would take the form of a prospective, parallel, randomized, active-comparator, unblinded interventional clinical trial.

The occurrence or non-occurrence of each safety and efficacy endpoint within the stated follow-up interval will be recorded. Potential confounders (subject age, gender, medical history, type and severity illness, type and duration of surgery, etc.) will also be recorded. These will be tested for balance between the two treatment groups by Student's t or chi-square tests. Variables found to be significantly unbalanced between groups (p ≤ 0.1) will be adjusted for in the analyses, as described below.

Statistical analysis will consist of a comparison, between treatment groups, of the fraction of subjects experiencing each of the two co-primary endpoints.

If **no **adjustment for potential confounders is necessary, the rates will be compared by cross-tabulating, for each endpoint, the treatment group by the occurrence/non-occurrence of the endpoint in a 2-by-2 table, and determining the "raw " (unadjusted) odds ratio for the occurrence of the event, for the diet, relative to the anticoagulant, along with its upper 1-sided 95% confidence limit. If adjustment for potential confounders **is **necessary, the odds ratio and its upper 1-sided 95% confidence limit will be obtained by multivariate logistic regression, where the occurrence of the endpoint is the dependent variable, the treatment group is the main independent variable, and the confounders are covariates. Alternatively, a *propensity score *can be calculated for each subject, incorporating all significant confounders. This score can be used in place of the individual confounders in the logistic regression.

Regardless of which way the odds ratios are calculated, significantly **better safety **for the diet will be inferred if the confidence interval around the odds ratio (diet/anticoagulant) for the primary safety endpoint lies entirely below the value of 1. **Non-inferior efficacy **for the diet will be inferred if the confidence interval around the odds ratio (diet/anticoagulant) for the primary efficacy endpoint lies entirely below the value 1.5, corresponding to an "allowable non-inferiority tolerance " of 50%.

The sample size required for 80% power to infer improved safety and non-inferior efficacy simultaneously will depend on the expected incidence rates of the primary safety and efficacy endpoints, the expected amount of improvement in safety for diet relative to anticoagulants, and the expected difference in efficacy (if any) between the two treatments.

While the FPE rate may appear to be the logical primary efficacy endpoint for RCTs comparing standard anticoagulant prophylaxis and treatment with a low VTE risk diet, FPE data are unreliable for several reasons:

• Autopsy rates have been low in recent years, so trial data and conclusions would rely on clinical diagnoses of FPE.

• Clinician FPE diagnoses correlate very poorly with autopsy diagnoses of FPE in both directions (i.e., more than 50% false positives and more than 50% false negatives [85]).

• At autopsy, the pathologist has to decide if the PE was the fatal event or just a contributing condition. This is quite subjective.

◦ Studies in the literature report postmortem exams inconsistently (i.e., classifications such as "incidental," "contributing to death" or "fatal" PE vary).

◦ In a meta-analysis of cohort studies with a low proportion of deaths investigated with autopsies, the overall mortality of patients undergoing elective hip and knee replacement was about 2 1/2 times the rate of FPE (total mortality: 0.93% versus FPE rate: 0.36%) with most of the non FPE deaths from heart failure or myocardial infarction [86]. FPE very frequently occurs in those with underlying severe cardiovascular disease, so FPE could not be ruled out without autopsies on all cardiovascular deaths.

• Using FPE at the sole primary endpoint takes no account of deaths from anticoagulant-induced bleeding.

Consequently, 'total mortality' should be the primary efficacy endpoint, because it is more reliable, consequential, and frequent than FPE. Screening for asymptomatic PE or DVT as efficacy endpoints would not be necessary, because they correlate poorly with FPE and total mortality.

The most consequential and frequent primary safety endpoint for RCTs would be 'total bleeding'. This includes minor bleeding, wound hematomas, and major bleeding.

Secondary endpoints should be FPE, symptomatic PE, symptomatic DVT, fatal bleeding, and major bleeding.

### Example--RCT for VTE prophylaxis of hospitalized medical inpatients: Low VTE risk diet versus standard anticoagulants

For a hypothetical example anticoagulant prophylaxis RCTs, consider acutely ill patients admitted to hospital medical wards. American Association of Chest Physician (ACCP) Guidelines suggest that such patients receive LMWH, low dose heparin, or fondaparinux as VTE prophylaxis^11^. We may use total bleeding (minor, major, and fatal bleeding) as the primary safety endpoint. A recent Cochrane Review of prophylactic anticoagulation for medical patients found that bleeding occurred in 117/2,405 (4.9%) of anticoagulated patients and 62/2,404 (2.6%) of untreated patients [87].

Subjects would be recruited from patients admitted to acute medical services who have VTE risk factors qualifying them for anticoagulant prophylaxis according to AACP Guidelines [11].

Subjects meeting the inclusion/exclusion criteria would be randomized to receive either a standard anticoagulant per AACP Guidelines (i.e., LMWH, low dose heparin, or fondaparinux) or a low-VTE-risk diet regimen. The diet (Mediterranean, vegetarian or vegan per the patient's choice) would be recommended for the entire duration of the trial, but compliance would be left to the discretion of the patient and treating physician. This flexibility in the diet arm assures that the diet intervention would not be a burden to patients. Flexibility in administering the low VTE diet is reasonable, since the RCTs are in large part testing the safety and efficacy of standard anticoagulation prophylaxis and treatment, and the low VTE diet would not be expected to do harm.

All outcomes will be recorded into the patients' medical records. In addition, follow-up RCT-related visits or calls as appropriate will be conducted approximately every 10 days up to 60 days after anticoagulation prophylaxis is completed, to determine if any of the primary or secondary safety or efficacy outcomes had occurred. Compliance with the low VTE risk diet will also be assessed, asking each patient to estimate his/her compliance on a scale of 0 - 100, with '0' representing completely non-compliant and '100' representing completely compliant. If follow-up takes place **before **discharge from the hospital, the information will be entered into the patient's medical record and separate data collection forms; if the follow-up takes place **after **discharge, the information will be recorded on to separate data collection forms.

The data collection forms will be assessed by study monitors at each participating medical center and reviewed by the responsible investigator at each medical center before anonymized data are sent to the study PI.

As noted above, we would use the overall death rate as the primary efficacy endpoint. Since the aforementioned Cochrane Review showed that prophylactic anticoagulation does not significantly reduce FPE rates or overall deaths in acute medical patients (for anticoagulation versus placebo, FPE: 0.94 [0.82, 1.07] and overall deaths: RR 0.95 [0.83, 1.07] [87]), a 50% non-inferiority tolerance is reasonable. The death rate in this Cochrane review was about 5% with or without anticoagulant prophylaxis. So with anticoagulant prophylaxis, the primary safety endpoint (overall bleeding) and the primary efficacy endpoint (overall mortality) would each be expected to occur in about 5% of patients. Expecting that the absence of anticoagulation and a low VTE diet can reduce the incidence of the total bleeding safety endpoint down to 2.5% with no change in efficacy, about 1,400 subjects would be required in each treatment group in order to have 80% power to meet both co-primary hypotheses (superior safety and non-inferior efficacy).

## Conclusion

Because of the bleeding and 'rebound hypercoagulability' risks of anticoagulation for VTE prophylaxis and the possibility that a low VTE risk diet might reduce the incidence of hospital-acquired VTE; randomized controlled non-inferiority clinical trials should be undertaken to compare standard anticoagulant treatment with a potentially low VTE risk diet (vegetarian, vegan, or Mediterranean) for VTE prophylaxis of medical and surgical patients. Likewise for VTE treatment, a randomized non-inferiority clinical trial comparing standard anticoagulation treatment with a low VTE risk diet should also be considered. We call upon the U. S. National Institutes of Health and the British National Institute for Health and Clinical Excellence to design and fund those trials.

## Competing interests

DKC withdrew warfarin from a patient with lower-limb deep venous thrombosis, disseminated tuberculosis, alcoholism, liver failure, and anemia because the risk for bleeding in this patient seemed greater than the benefit of anticoagulant treatment. The patient later died of pulmonary embolism. DKC lost his medical license because of this case, having had no other medical board discipline during 25 years of clinical practice. Otherwise, the authors declare that they have no competing interests.

## Authors' contributions

DKC proposed the concept of dietary prophylaxis for VTE and a non-inferiority trial for comparison with anticoagulants, and reviewed the relevant literature. PCM was primarily responsible for formulating the VCHH and evaluating its relevance to VTE prophylaxis and treatment. PSA investigated the molecular aspects of the VCHH and their consistency with the dietary hypothesis of prophylaxis. JCP was responsible for the statistical considerations and the design of the proposed non-inferiority trial. All authors read and agreed the final manuscript.
